# Resilience among family caregivers of children and adolescents followed for diabetes

**DOI:** 10.1192/j.eurpsy.2025.1599

**Published:** 2025-08-26

**Authors:** S. Kolsi, W. Abbes, A. W. Noureddine, A. Bouaziz, Y. Ben Ayed, K. Medhaffar, K. Zitoun

**Affiliations:** 1 Psychiatry, Hospital University, Gabes, Tunisia

## Abstract

**Introduction:**

Diabetes is a real public health problem. Some people seem to be more vulnerable to this pathology, especially children and teenagers. To ensure their loved one’s well-being, caregivers can also develop coping strategies.

**Objectives:**

- Assessing caregivers’ resilience of children and adolescents with diabetes.
- Identify factors associated with a high level of resilience.

**Methods:**

The study was conducted at the University Hospital of Gabès, in the pediatrics and internal medicine departments, as well as in outpatient clinics with caregivers of children and adolescents during the period from March 2024 to May 2024.

The sociodemographic and clinical characteristics of caregivers of children and adolescents have been collected using a pre-established form.

We used :

The resilience scale (CD-RISC) is designed to measure resilience, defined as the ability to cope with traumatic events. The higher the score, the greater the resilience.

**Results:**

Our sample included 32 caregivers. The mean age was 35.55 years with extremes of 22 and 55 years and the sex ratio (M/F) was 0.6.

Among the participants, the majority, 78.1% (n=25), presented symptoms of psychological distress. A high level of stress was perceived by 43.7% of caregivers and satisfaction was noted by 34.4%.

The mean resilience score was 59.06 (SD =10.45) with extremes of 39 and 81.

We found a significant correlation between a high level of resilience and symptoms of psychological distress (p=0.003), the level of daily stress (p=0.03) and the level of overall satisfaction (p=0.043).

**Conclusions:**

Our study showed that a high level of resilience is linked to several factors.

Interventions targeting stress related to social events should be integrated to increase the caregivers’resilience.

**Disclosure of Interest:**

None Declared

